# Fertile *Goeppertella* from the Jurassic of Patagonia: mosaic evolution in the Dipteridaceae-Matoniaceae lineage

**DOI:** 10.1093/aobpla/plad007

**Published:** 2023-06-07

**Authors:** Agustina Yañez, Ignacio H Escapa, Thereis Y S Choo

**Affiliations:** Museo Argentino de Ciencias Naturales ‘Bernardino Rivadavia’, CONICET, Av. Ángel Gallardo 470, Ciudad de Buenos Aires, Argentina; Consejo Nacional de Investigaciones Científicas y Técnicas, Museo Paleontológico Egidio Feruglio, Trelew, Chubut 9100, Argentina; Singapore Botanic Gardens, National Parks Board, Singapore

**Keywords:** Evolution, Gondwana, Gleicheniales, *Goeppertella unicyclica*, Jurassic, Patagonia, reproductive traits

## Abstract

*Goeppertella* has been postulated as a monophyletic group, whose precise position within the Gleichenoid families Dipteriaceae and Matoniaceae, remains poorly understood. Previously described *Goeppertella* specimens are based on frond fragments and its fertile morphology is represented by a few, poorly preserved specimens. We describe a new species based on the largest collection of fertile specimens known to date and discuss the evolutionary history of the genus based on the additional reproductive characters provided by the fossils described. Plant impressions were collected in Early Jurassic sediments of Patagonia, Argentina. The specimens were described, and silicone rubber casts were developed to examine in detail vegetative and reproductive features. The new species was compared with other *Goeppertella* species. Finally, a backbone analysis was performed in the context of a previously published combined matrix of Dipteridaceae, using the maximum parsimony criterion. The new species is described based on a combination of features that have not been previously reported. The vegetative morphology shows affinities with most fossil and extant Dipteriaceae, contrasting with the reproductive morphology which is more comparable with the scarce number of fossil dipteridaceous forms and it is more spread in the sister family, Matoniaceae. The backbone analysis indicates that the position of the new species vary among different positions among Dipteridaceae and Matoniaceae. Additional analyses, discriminating the signal of reproductive and vegetative character, are provided to discuss the base of this uncertainty. We consider *Goeppertella* as a member of the family Dipteridaceae since we interpret most shared features with Matoniaceae as plesiomorphic conditions for the family. In contrast, most shared features with Dipteridaceae represent apomorphies for the group. Thus, *Goeppertella* would represent an early diverging genus in Dipteridaceae, considering the venation characters as the most important in order to define the family.

## Teaser

We describe a new fern fossil species of Goeppertella (Dipteridaceae) based on the largest collection of fertile specimens known to date. From phylogenetic analysis using vegetative and reproductive traits separately, we discuss the evolutionary history of the genus, its relevance to understand the early evolution of the family and the affinity with the sister clade, Matoniaceae.

## Introduction

Gleichenoid ferns (Gleicheniales order; Christenhusz *et al.*, 2011; Christenhusz and Chase, 2014; PPG I, 2016) originated during the Permian, approximately 270 ma ago, and is one of the sister group of the remaining leptosporangiate ferns, includes the majority of extant lineages (Pryer *et al*., 2004; Testo and Sundue, 2016). This clade quickly diversified during the late Paleozoic (Schuettpelz and Pryer, 2009) and today is composed of three families: Gleicheniaceae, the earliest diverging family within the group, followed by sister families, Matoniaceae and Dipteridaceae (Pryer *et al*., 2004). The monophyly of Gleicheniales has been often questioned based on different partitions of molecular data (Qi *et al*., 2018 and citations therein; Shu *et al*. 2022). However, from a morphological point of view, members of Gleicheniales share the unique characters of having fronds with a branching main rachis, resulting in the frond having usually 2, but sometimes more, axes of growth (Stevenson & Loconte, 1996; Moran, 2019). Additionally, other synapomorphies have been reported, such as root steles with 3–5 protoxylem poles (Schneider 1996) and antheridia with 6–12 narrow, twisted, or curved cells in walls (Smith *et al*. 2006). In relation to reproductive structures, Millay and Taylor (1990) have pointed out that ‘the close similarity in sorus and sporangium morphology between the Matoniaceae, Dipteridaceae and Gleicheniaceae which presumably indicates derivation from a common stem group’, although they did not explain what these similarities are.

The family Dipteridaceae is represented in modern-day flora by two small genera, *Cheiropleuria* and *Dipteris*, with a total estimated number of 11 species (PPG I, 2016). These extant Dipteridaceae species have a restricted distribution and are found only in the warm tropics of the Asia-Pacific region (Kato *et al.*, 2001) on streambanks where the canopy is open, or colonizing disturbed sites and exposed ridges (Holttum, 1954; Kramer, 1990). In contrast, this family constituted one of the predominant elements in the warm-temperate and subtropical regions of the world during the Mesozoic (Tidwell and Ash, 1994; Zhou *et al*., 2016). Based on a combined phylogenetic analyses, Choo and Escapa (2018) recognized five monophyletic extinct genera: *Clathropteris* Brongniart, *Digitopteris* C. Pott and Bomfleur, *Goeppertella* Oishi and Yamasita*, Sewardalea* Choo and Escapa and *Thaumatopteris* Goeppert, and two unnatural and unresolved groups: *Dictyophyllum* Lindley and Hutton and *Hausmannia* Dunker (Oishi and Yamasita, 1936; Van Konijnenburg-van Cittert, 2002; Choo and Escapa, 2018). More recently, Gnaedinger and Zavattieri (2021) described a new genus and species, *Patagoniapteris artabeae* from Late Triassic of Neuquén, that would represent a transitional form between fossil genera and extant *Dipteris* species.

The oldest records of the family date back to the Middle Triassic of both the Northern Hemisphere, represented by *Clathropteris* (Kustatscher and Van Konijnenburg-van Cittert, 2011) and the Southern Hemisphere, represented by *Thaumatopteris*, *Dictiophyllum* and *Hausmannia* (Webb, 1982; Bodnar *et al*. 2018). Later, towards the Upper Triassic and Lower Jurassic, the family shows an increase in richness and diversity (Tidwell and Ash, 1994; Rees and Cleal, 2004). Dipteridaceae records decrease significantly globally from the late Jurassic and throughout the Late Cretaceous, being almost exclusively represented by the genus *Hausmannia* (Feruglio, 1937; Cantrill, 1995; Stockey *et al.*, 2006; Golovneva and Grabovskiy, 2019).

All Dipteridaceae share the synapomorphy of having highly reticulated venation, however the gross frond morphology of Mesozoic genera varies significantly in the number, disposition, and morphology of primary frond segments. These genera includes representatives with entire or irregularly segmented fronds (e.g. *Hausmannia* and *Clathropteris*), once pinnate (e.g. *Camptopteris*, *Dictiophyllum*, *Patagoniapteris*, and *Thaumatopteris*) or, apparently, twice pinnate (Rees, 1993) fronds. This last condition is exclusive to the genus *Goeppertella*, first described by Oishi and Yamasita (1936) based on observations from Schenk (1867) and Zeiller (1903) on specimens identified as *Woodwardites microlobus* from the Late Triassic (Rhaetian). Altogether, ca. 20 species of *Goeppertella* have been described from across the globe and spanning the Late Triassic to Jurassic (Zhou *et al*., 2016). In the Southern Hemisphere, *Goeppertella* is only known from a few occurrences in the Late Triassic (Herbst, 1993, 2000; Morel *et al*., 1999), with most of the diversity collected in Jurassic sediments of South America (Herbst, 2000), Antarctica (Rees and Cleal, 2004) and New Zealand (Rees, 1993).

From a phylogenetic point of view, the precise *Goeppertella* position within the Dipteriaceae and the Matoniaceae remains poorly understood. Because all described *Goeppertella* specimens are based on frond fragments, and therefore the complete architecture of the frond is only hypothetized (Rees, 1993), on phylogenetic study developed by Choo and Escapa (2018) the genus it was only reconstructed as a monophyletic clade in the base of Dipteridaceae when it was scored according to the aforementioned hypothetical architecture. In the same way to the frond architecture knowledge, the fertile morphology of the genus is represented for a few, poorly preserved specimens, which fail to show details in soral and sporangial morphology, distribution and development.

In the present work, we describe a new species for the genus *Goeppertella*, based on numerous sterile and fertile specimens from the Early Jurassic Cerro Bayo locality (Patagonia, Argentina. Escapa *et al*., 2008). Exquisitely preserved reproductive features reveal close similarities of *Goeppertella* with Dipteridaceae, but also with the sister family Matoniaceae. We provide an analysis of the phylogenetic circumscription of the genus based on the additional characters provided by the fossils described here, including the implications of the findings regarding the organization of the sori and sporangia. Furthermore, we discussed the basis of the instability of *Goeppertella* in the light of the new information presented here.

## Materials and Methods

### Geologic setting and paleobotanical context

The Cerro Bayo locality is situated near Gastre in the northwest part of Chubut Province, Argentina (Escapa *et al*., 2014). Plant horizons at this locality belong to an unnamed unit of fluvially reworked, volcaniclastic deposits, which are overlain by volcanic and volcaniclastic deposits of the Lonco Trapial Formation. Radiometric studies have restricted the age of the site to the Early Jurassic (most likely Pliensbachian) (Cúneo *et al.*, 2013; Escapa *et al*., 2014; Figari *et al*., 2015). From a chronostratigraphic perspective, Cerro Bayo is considered to be an approximately lateral equivalent of Las Leoneras formation, which crops out around 80 km southeast of Cerro Bayo (Nakayama, 1973). The early sauropodomorph *Leonerasaurus taquetrensis* is the single fossil so far reported from Las Leoneras (Pol *et al*., 2011), but plants mega or microfossils are so far unknown for the unit.

**Figure 1.**
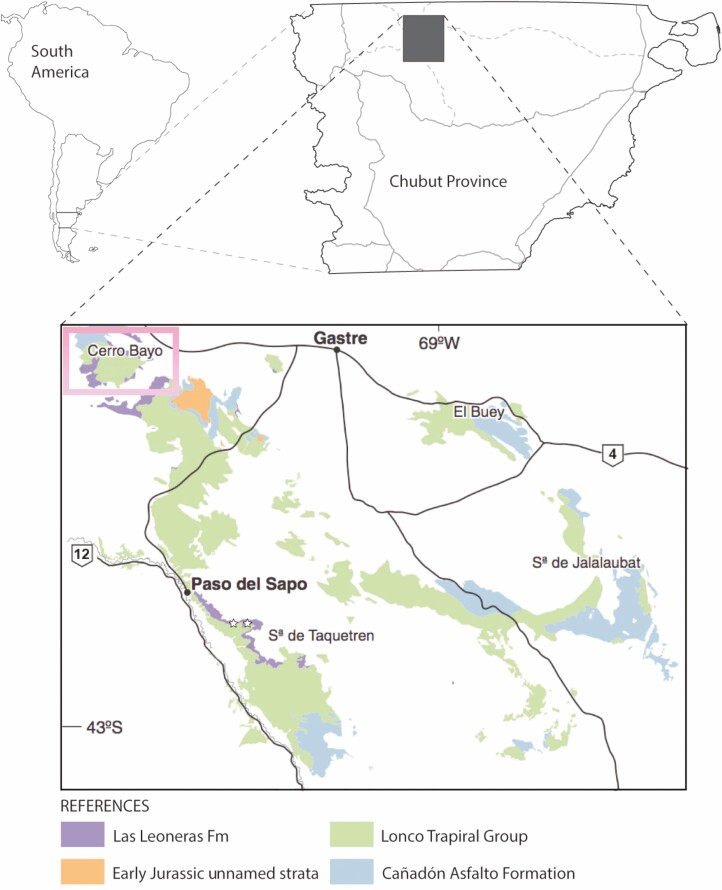
Location and geology of the Cerro Bayo Area, where *Goeppertella unicyclica* specimens were collected (GPS coordinates available upon request). Geologic map modified from Cúneo *et al.* (2013).

Specimens were collected at three nearby quarries (GPS coordinates are available upon request to the authors) in the general Cerro Bayo locality (Fig. 1). Plant impressions occur in fine-grained, partially silicified sediments as part of a taphocoenosis including largely dominated by conifers and ferns. The most conspicuous elements in this flora include species within Equisetaceae (Elgorriaga *et al*., 2015), Osmundaceae (*Todites cacereii* and *Osmundopsis rafaelii*; Escapa & Cúneo 2012), Marattiaceae (Escapa *et al*., 2014), Dipteridaceae (*Clathrotpteris meniscoides*; Choo *et al.*, 2016) and Cupressaceae (*Austrohamia minuta*; Escapa *et al*. 2008; Bodnar and Escapa, 2016). Putative an dicksoniace vegetative fronds and seed fern leaves (Caytoniales; Elgorriaga *et al.*, 2019) complete the floral spectrum.

### Fossil preparation and illustration

The *Goeppertella* specimens described here are housed in the Museo Paleontológico Egidio Feruglio Palaleobotanical Collection in Trelew, Argentina (hereafter MPEF-Pb). Photographs of specimens were taken with a Canon EOS 7D camera equipped with a Canon EF-S 60 mm macro lens; additional extension tubes were used in order to obtain higher magnifications. In order to obtain greater depth of focus for high-magnification images, we used an image-stacking technique (Bercovici *et al*., 2009) in which one well-focused image is obtained via merging several individual photographs captured at slightly different focal planes. Images were checked and edited for the presence of artefacts related to the stacking process, edited for colour balance and cropped for publication using Adobe Photoshop.

Most reproductive structures in the specimens described here are preserved as moulds. Therefore, making examinations and illustrations of detailed morphological features of the sori of *Goeppertella* required the development of silicone rubber casts (Watson and Alvin, 1976). Details of the technique were discussed by Escapa *et al*. (2014) when applied to the synangia of *Marattiopsis patagonica*, also from the Cerro Bayo locality. Silicone rubber casts were also explored using SEM Philips XL 30 TMP New Look from the Microscopy Service of Museo Argentino de Ciencias Naturales ‘Bernardino Rivadavia’ (Buenos Aires City).

### Morphological comparisons

In order to identify, describe and differentiate studied specimens from other *Goeppertella* species, we carried out a detailed morphological comparison. We selected all species that were described for the southern hemisphere, as well as the best representative species of the Northern Hemisphere **[see****Supporting Information 2 and 3****]**.

### Terminology

The knowledge of the frond architecture of *Goeppertella* is highly fragmentary. In particular, the presence of a basal dichotomy generating two rachial arms is generally assumed, but it has not been described and illustrated. Consequently, different terms have been proposed to describe and hypothetically reconstruct the complex architecture that characterizes the fronds (Oishi and Yamasita, 1936; Holttum, 1954; Arrondo and Petriella, 1982).

**Figure 2.**
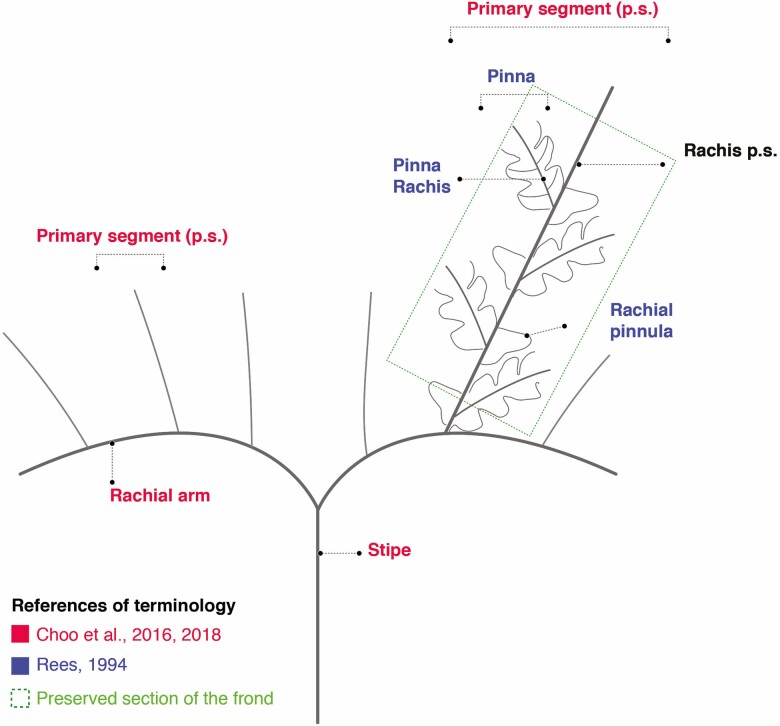
Schematic representation of *Goeppertela* showing terminology adopted in the present work.

Recently, Choo and Escapa (2018) adopted a set of descriptive terms, which can be used consistently across different Dipteridaceae forms. In this work, the last scheme will be followed to refer to the first orders of frond division. In addition, because we accept the hypothesis that *Goeppertella* has bipinnate fronds, the terminology of Rees (1993) will be adopted to describe the last orders of division (Fig. 2). Additionally, current fern terminology was used to describe the shape of some parts of the frond (Lellinger, 2002).

We follow the taxonomic scheme proposed by PPG I (2016).

### Phylogenetic analysis

We conducted two main analyses to discuss the phylogenetic position of *Goeppertella*.

First, we conducted a maximum parsimony analysis using the slightly modified taxon sampling of Choo and Escapa (2018) to evaluate phylogenetic relationships of the new fossil in the Dipteridaceae context, and obtaining similar results in terms of the instability of fossil species **[see****Supporting Information 1****]**.

In this context, we focused on further analyse the affinity of *Goeppertella* with Matoniaceae and Dipteridaceae, considering the vegetative morphology, and the reproductive morphology described here. For this, we used a morphological submatrix of the one published by Choo and Escapa (2018) in which only the extant representatives were included, using the topology obtained in that combined study, as a backbone. *Goeppertella unyciclica* was scored in the context of the morphological matrix, which includes a total of 51 characters, of which 31 correspond to vegetative structures and 20 to reproductive structures (Appendix 1). All characters correspond to those used by Choo and Escapa (2018), except the N°23 that was added for the present analysis in order to analyze the evolution of the type of venation through the evolution of the order. Some character states were rescored from the original matrix based on reinterpretations and new available data.

The optimal and suboptimal positions of *Goeppertella* were determined by manually editing the backbone in TNT (Goloboff and Catalano, 2016) to move the genera to different positions, and recording the score of the tree (affinity analysis). Likewise, the maximum parsimony analysis was repeated for the vegetative and reproductive traits separately, to discuss the origin of the uncertainty in the evolutionary position of the genus. All the analyses were conducted using TNT.

## Results

### Systematics

#### Class:

Polypodiopsida Cronquist, Takht. and W.Zimm.

#### Order:

Gleicheniales Schimp.

#### Family:

Dipteridaceae Seward and Dale

#### Genus:

*Goeppertella* Oishi and Yamasita, 1936

#### Type species:

*Goeppertella microloba* (Schenk) Oishi and Yamasita, 1936

**Figure 3.**
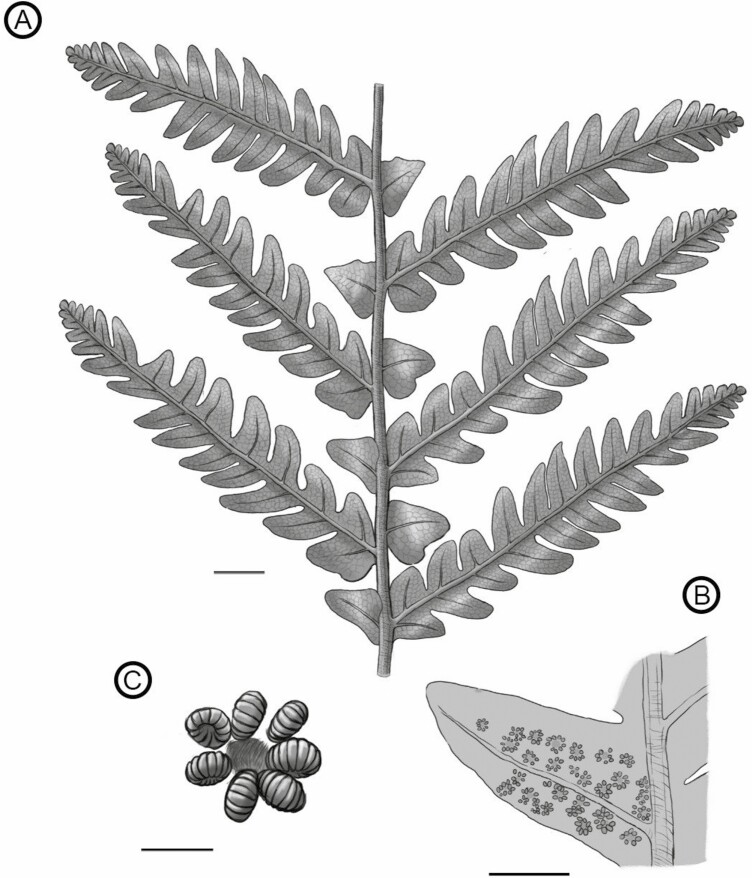
Hypothetical reconstruction of *Goeppertella unicyclica*. (A) Fragment of primary segment; (B) Detail of fertil pinnula; (C) Detail of sorus. Scale bars = (A) 1 cm, (B) 5 mm, (C) 1 mm.

**Figure 4.**
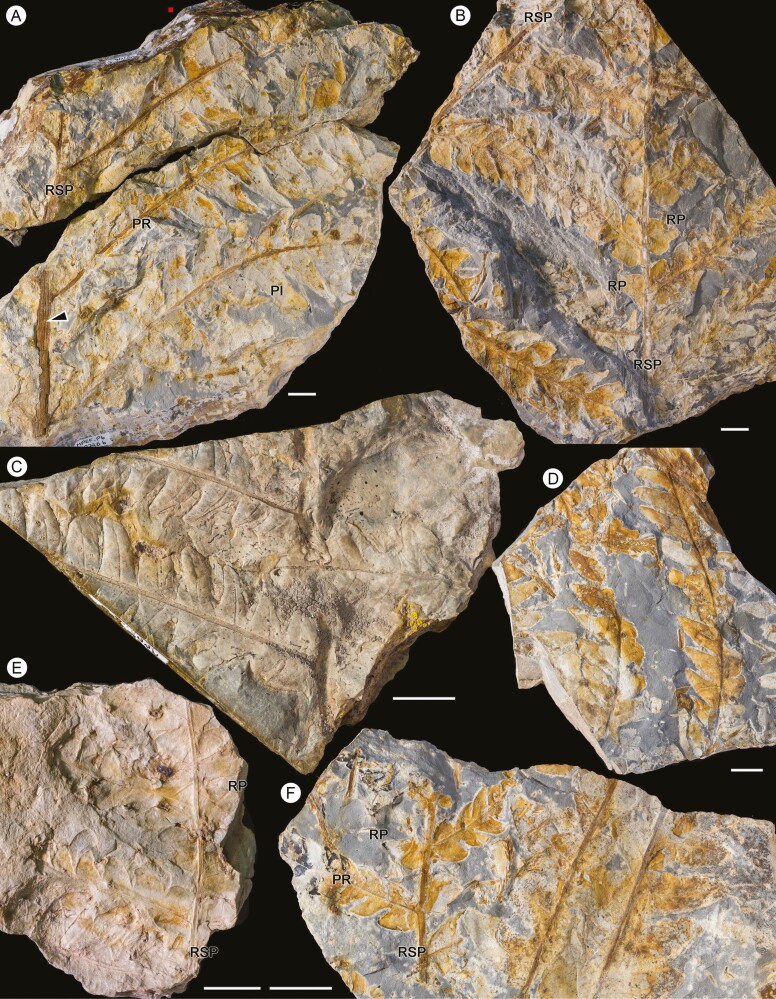
Fragments of primary segments 1-pinnate pinnatifid, bearing pinnatifid to pinnatisect, linear pinnae, and sessile, deltate to auriculate rachial pinnules; in (A) the rachis of primary segment shows longitudinal ribs (arrowhead). Scale bars = 1 cm. Abbreviations: Pl, Pinnule; PR, pinna rachis; RP, rachial pinnula; RSP, rachis of primary segment. (A) MPEF-Pb 2746a and 2746b, (B) MPEF-Pb 2707, (C) MPEF-Pb 6965 (D), MPEF-Pb 2734, (E) MPEF-Pb 6925, (F) MPEF-Pb 2754.

**Figure 5.**
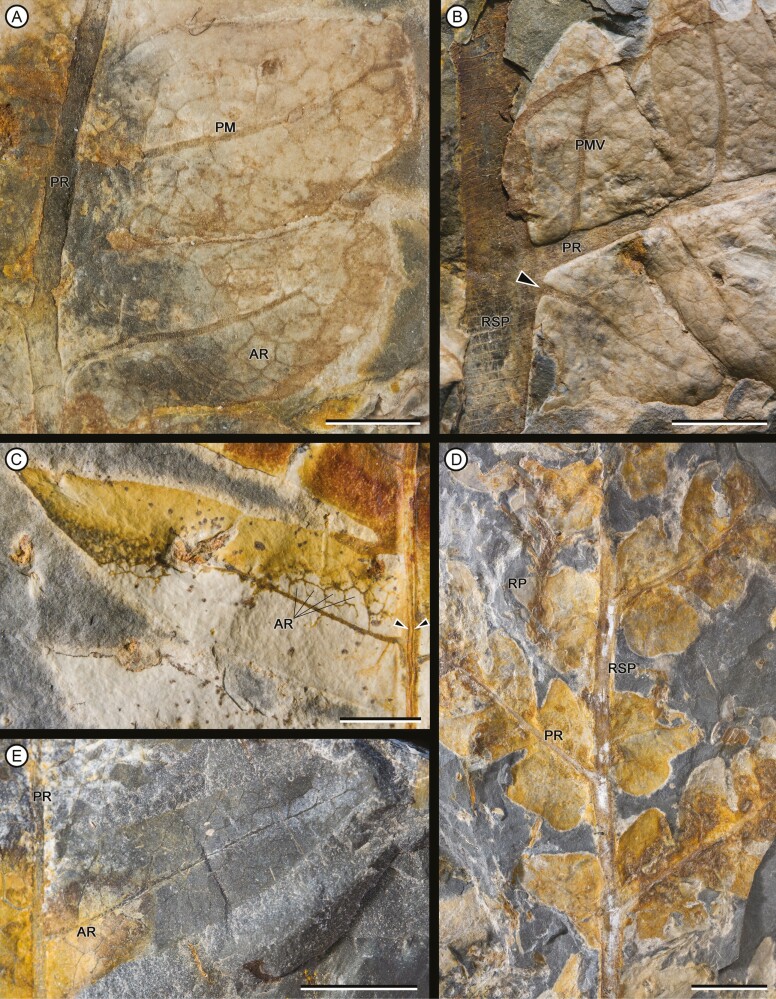
(A) Pinnules showing its insertion in the pinna rachis, with a midvein thick, becoming finer towards the apex. MPEF-Pb 6575. (B) detail of the pinna base showing its insertion in the primary segment. The midvein of the pinnula originates from the latter (arrowhead). MPEF-Pb 6575. (C) pinnula showing first-order polygonal areole. Longitudinal ribs are observed on pinna rachis (arrowheads). MPEF-Pb 11062. (D) fragments of primary segments bearing intercalated pinnae and rachial pinnulae. MPEF-Pb 2707. (E) detail of a pinnula showing areolate venation. MPEF-Pb 6837. Scale bars = (A-C, E) 5 mm; (D) = 1 cm. Abbreviations: AR, areole; PMV, pinnule midvein; RP, rachial pinnula; PR, pinna rachis.

**Figure 6.**
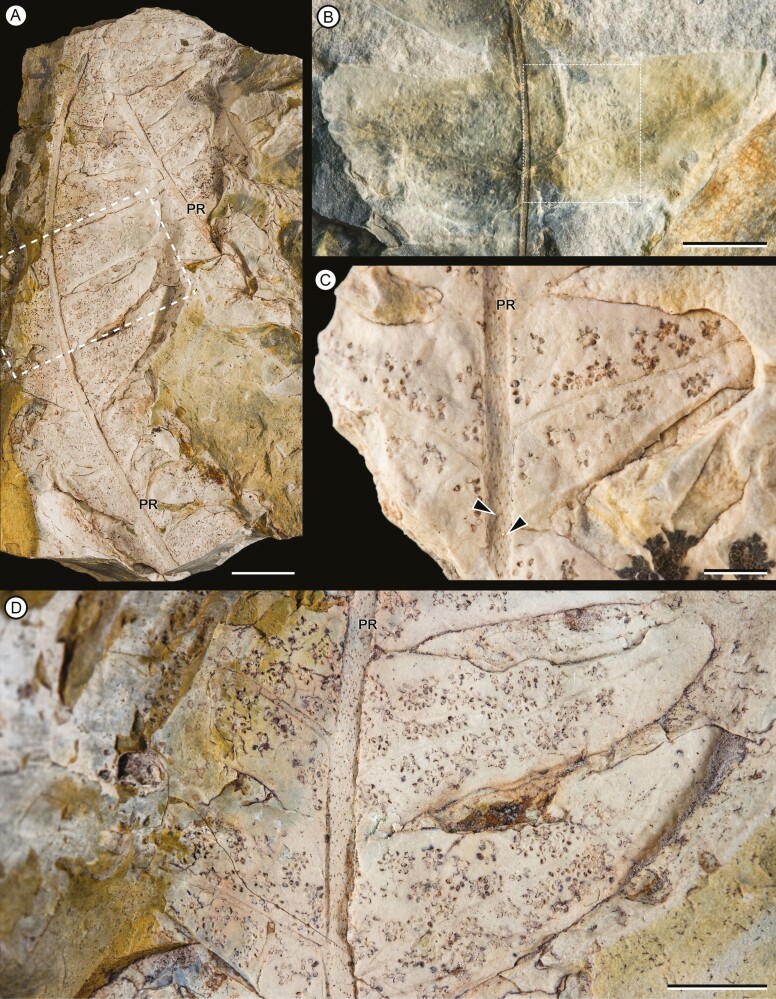
(A) Fragments of fertil pinnae. The area included in the dotted margin box corresponds to image D. MPEF-Pb 3573. (B) fragments of pinnae showing two fertile pinnulae. Sori are distributed on the basal portion of the pinnula (dotted margin box) suggesting apparent acroscopic maturation within the pinnula. MPEF-Pb 6939. (C) fragments of two fertile pinnulae showing round sori, alone or partially overlapping. Pinna rachis is covered by trichome bases (arrowheads). MPEF-Pb 6573. (D) detail of image A showing two fertil pinnulae, bearing partially overlapping sori. MPEF-Pb 3573. Scale bars = (A) 1 cm, (B, D) 5 mm, (C) 2 mm. Abbreviations: PR, pinna rachis.

**Figure 7.**
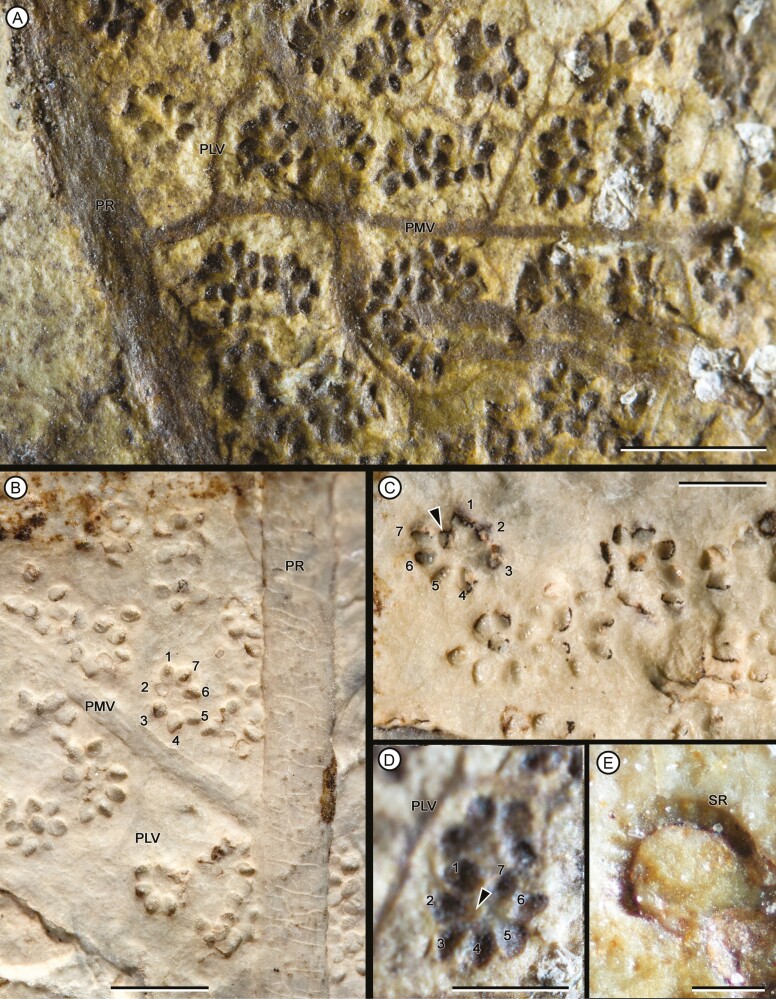
Detail of fertile pinnula. (A) Circular to slightly elongated sori, partially overlapping, borne inside of areolae. MPEF-Pb 11117. (B, C) Sori are arranged on both sides of the midvein and composed by seven-eight sporangia (numbers). MPEF-Pb 6939. (D) sporangia borns around an insertion area (arrowheads). MPEF-Pb 11117 (E) sporangia are leptosporangium type with oblique dehiscence ring. MPEF-Pb 6927. Scale bars = (A-C) 2 mm, (D) 1 mm, (E) 0.1. Abbreviations: PLV, pinnule lateral vein; PMV, pinnule midvein; PR, pinna rachis; SR, sporangial ring.

**Figure 8.**
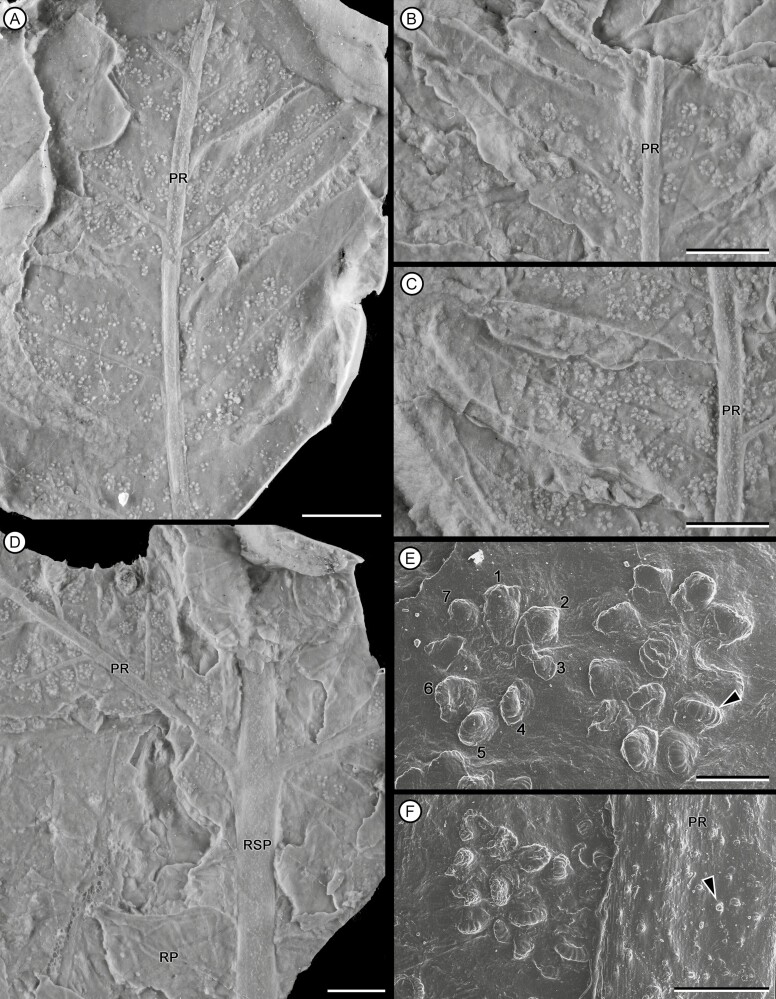
Silicone rubber casts of fertile fragments of primary segments and pinnae (A) Fragment of fertile pinna. MPEF-Pb 3573. (B) apparently early stages of maturation of the fertile pinna showing isolated sori. MPEF-Pb 3573. (C) apparently later stages of maturation of the fertile pinna showing partially overlapping sori. MPEF-Pb 3573. (D) Fragment of primary segment showing the insertion area of fertil pinnae and rachial pinnula. MPEF-Pb 3573. (E) a sorus (left) and two sori partially overlapping (right); leptosporangium with oblique dehiscence ring occupying ¾ parts of the sporangium circumference (arrowhead). MPEF-Pb 6939. (F) partially overlapping sorus next to pinna rachis; pinna rachis showing trichome bases (arrowhead). MPEF-Pb 3573. Scale bars = (A-D) 5 mm, (E) 0.05 mm, (F) 0.01 mm. Abbreviations: PR, pinna rachis; RP, rachial pinnula; RSP, rachis of primary segment.

*Goeppertella unicyclica* Escapa and Yañez sp. nov. (Figs. 3–8)

#### Diagnosis:

Primary segments 1-pinnate-pinnatifid with a thick and grooved rachis. Pinnae linear or lanceolate, sessile, varying between subopposite and alternate. Pinnules partially fused, sessile, lanced to triangular-falcate, with acute apex and entire margins; the basalmost one borne on the basiscopic side of the pinna and irrigated from the primary rachis. Rachial pinnules solitary, borne subopposite and spaced between pinnae, deltate, the basiscopic side more developed, forming auricles. Axes and laminar tissue covered by trichome bases. Circular to slightly elongated sori comprising a uniseriate row of leptosporangia, around a well-defined area of insertion.

#### Etymology:

The specific epithet refers to the sori with a uniseriate row of sporangia, which is characteristic of the species.

#### Holotype:

MPEF-Pb 6573; *Paratypes* MPEF-Pb 1570, 2706, 2707, 2716, 2719a, 2719b, 2734, 2737b, 2746a, 2746b, 2754, 2767, 6572, 6573, 6575, 6595, 6812, 6818, 6826, 6837, 6843, 6904, 6906, 6922, 6925, 6927, 6931, 6935, 6939, 6940, 6942, 6950, 6955, 6965, 6995, 6840, 11062, 11117, 16563.

#### Repository:

Museo Paleontológico Egidio Feruglio paleobotany collection (MPEF-Pb), Trelew, Chubut, Argentina.

#### Type locality:

Cerro Bayo, Chubut, Argentina.

#### Stratigraphic position and age:

Unnamed stratigraphic unit, lateral equivalent of Las Leoneras Formation; early Jurassic (Pliensbachian).

#### Description:

Overall form of frond, primary segments and pinnae unknown. Fragments of primary segments 1-pinnate-pinnatifid, up to at least 8.66 cm wide (Fig. 4). Rachis of primary segments, right, up to at least 16.3 cm length and 2.26–4.75 mm wide, tapering in apical direction, with at least three longitudinal ribs (Fig. 4A), and scattered trichome bases. Pinnae borne laterally in one plane, sessile, varying between subopposite and alternate, at intervals of 9.82–18 mm (between consecutive pairs of pinnae) and at angles of 52°–79° to the rachis of primary segments, length at least 14.8 cm and wide ranging from 1.44 to 5.01 cm, equally wide at the base and middle, apparently linear in shape (a single specimen presents a lanceolate pinna, with a reduction in width towards the apex: MPEF-Pb-2706), pinnatifid to pinnatisect, incised up to 3/4 or more of the width. Pinna rachis up to at least 0.29–2.4 mm wide, right proximally, curved in the middle towards the base of primary segments (Fig. 4A), with longitudinal ribs (Fig. 5C) and densely covered by trichome bases 0.1–0.5 mm apart, the round trichome bases 0.06–0.1 mm diam, or, in some specimens, elongated (MPEF-Pb-6965) (Fig. 8F). Basalmost pinnula occurring on basiscopic side of pinna, with primary vein borne from rachis of primary segments (MPEF-Pb-6575) (Fig. 5B). Pinnules sessile, inserted throughout its base, varying between opposite, subopposite or less frequently alternate, at intervals of 2.1–17.7 mm (measured between main veins of pinnules), borne at angles of 50°–81° to the pinna rachis, length (measured along main vein from pinnule apex to pinna rachis) ranging from 6.78 to 35.7 mm, the basal shortest except MPEF-Pb-2706, wide (between sinus point) ranging from 6.29 to 20.8 mm, the narrower pinnules tending to occur near the pinna apices, with a long and sharp or acuminate apex and an almost truncated base, wider near or at the base (i.e. lanceate) (Figs. 4, 5A, C, E), or occasionally, the first two pairs of segments being ovate (Fig. 5B, D), margin entire with trichomes (MPEF-Pb-1570), laminar tissue between veins with trichome bases. Rachial pinnules sessile, occupying the gap between each successive pair of pinnae, subopposite, borne at intervals of 9.97–20.73 mm and at angles of 46°-86° to the pinna rachis, length (measured along main vein from rachial pinnule apex to pinna rachis) ranging from 8.35 to 13.01 mm, wide (between sinus point) up to at least 14.17 mm, deltate, the basiscopic side more developed forming an auricle (Figs. 4B, 5D, 8D). Main veins of pinnules and rachial pinnules up to 0.2–0.7 mm wide, which runs to the apex and defines its long axis, straight or following a slightly sinous course, becoming finer towards the apex, densely and regularly covered by trichome bases (Fig. 5A, B); lateral veins only evident in some specimens (Fig. 7A), borne at intervals of about 2.1–3.9 mm, branched to produce a network of polygonal or rectangular areolae about 1.6 mm but sometimes elongated up to 2.8 mm long, twice as long as broad, at least five between the main vein and the margin of pinnule (Figs. 5A, C, E, 7A); within the areolas some specimens have a minor venation order while in others free veins appear to be seen (MPEF-Pb-2716, MPEF-Pb-2719, MPEF-Pb-2746a). Sori circular to slightly elongated, ranging from 0.9 to 1.1 mm in diameter, borne inside of areola, alone or in groups of up to three, sometimes partially overlapping (Figs. 6, 7, 8), apparent acroscopic maturation within pinnula; seven-eight sporangia per sorus, born around an insertion area of 0.3–0.8 in diameter (Figs. 7B–D, 8E, F); leptosporangium with capsule of about 0.2 mm in diameter, with oblique dehiscence annulus occupying 3/4 of the sporangium circumference, ring cells 0.03–0.05 mm wide (Figs. 7E, 8E). Most of the specimens with the annuli turned, the dehiscence seems to have started because some expanded annulus are observed. No pedicels were observed.

### Generic assignation

The specimens analyzed here show the anastomosed pattern of venation with polygonal areoles that characterizes the fossil and extant Dipteridaceae (Seward, 1900; Seward and Dale, 1901; Zeiller, 1903). It has been noted that the venation pattern of the different frond parts can be extremely similar among different Mesozoic genera and, therefore, highly fragmentary pieces can be difficult to assign at the genus level (Rees and Cleal, 2004; Escapa *et al*., 2008). Notwithstanding this, *G. unicyclica* matches the combination of diagnostic vegetative characteristics indicated in the early descriptions of the genus (Zeiller, 1903; Oishi and Yamasita 1936) such as the presence of (at least) bipinnate primary segments bearing partially fused pinnules and the presence of rachial pinnules between pinnae.

Regarding the reproductive characters, *G. unicyclica* is characterized by having circular to slightly elongated sori, arranged on both sides of the middle vein and borne inside the areoles, with each sori comprising a cycle of seven or eight leptosporangia. The sporangia are arranged in a uniseriate row around a well-defined, slightly concave area of insertion. They have capsules with an oblique dehiscence annulus in which cells with thickened walls are distinguished. Oishi and Yamasita (1936) established the genus *Goeppertella* after specimens previously described under the name *Woodwardites microlobus* (Schenk, 1867; Schimper, 1869; Zeiller, 1903). Despite its multiple occurrences around the north of Europe and southeast of Asia (Arrondo and Petriella, 1982), the descriptions of reproductive organs of this species are scarce and poorly illustrated. Based on specimens from the Triassic of Germany, Schimper (1869) defined the sori as ‘oblong and biseriate’ but the three drawings that illustrate the description are general views of the frond and the pinna, and do not explicitly show reproductive details. Based on a collection from the Triassic on Indochina, Zeiller (1903) mentioned that the sporangia cover the entire surface of the pinnule, and both fertile and sterile pinnules are morphologically similar. Although the illustrations are not conclusive, Zeiller described the sporangia as clustering in groups of 5-8, which if compared with the specimens described here, supports the idea that these groups also have unicyclic sori. A deeper review of this, and other collections of *Goeppertella microlobus*, are crucial in order to amend the genus to include details of sori organization and distribution.

### Comparisons

As explained in the following paragraphs, the *Goeppertella* specimens described here were assigned to a new species in the genus based on a combination of vegetative and reproductive features that have not been previously reported. It is important to consider, however, that the great majority of the known species have been described on the basis of poorly preserved, highly fragmentary and/or sterile specimens. In addition, most vegetative morphologic features show a high degree of variation among Dipteridaceae in general, and in *Goeppertella* in particular (Arrondo and Petriella, 1982; Herbst, 1992). In this context, it seems possible that further re-descriptions of previously known species may show more similarities to our specimens listed here, and therefore modifying the taxonomic decisions taken in this work.

The following section provides a comparison of the main vegetative and reproductive features described for *Goeppertella unicyclica* in reference to other described *Goeppertella* species. More extensive and detailed comparisons are included in **see****Supporting Information 3—Table S2**.

#### Vegetative morphology:

In their review of the genus, Arrondo and Petriella (1982) focused on the significance of the subsidiary elements (interpinnular leaf appendages) for the taxonomy of the group, recognizing two main types according to whether the origin of their venation was axillary or intercalary. *Goeppertella unicyclica* shows the latter condition (Fig. 3), where the venation arises directly from the rachis of the primary segment in structures also referred to as rachial pinnulae. The rachial pinnulae observed in the species described here resembles the rachial pinnulae described for *G. microloba* (Schenk, 1867; Zeiller, 1903; Cazaubon, 1947), *G. macroloba* (Herbst, 1964), *G. neuqueniana* (Herbst, 1966), *G. stipanicicii* (Herbst, 1992), *G. woodii* (Rees, 1993) and *G. taverai* (Herbst, 2000). Among these, a greater similarity was observed with *G. macroloba:* both species have rachial pinnules of similar dimensions and also share other additional features such as pinnules being lanced to triangular-falcate, with acute apices and entire margins.

A second feature of potential taxonomic relevance is the presence of a continuous laminar wing that is more or less parallel to the rachis and connects the pinnae to the rachial pinnula (i.e. rachial lamina) (Arrondo and Petriella, 1982; Rees 1993). This character is present in the species mentioned above but is absent in *Goeppertella unicyclica, G. frenguelliana* (Cazaubon, 1947) from Jurassic sediments of the Esquel Range in Chubut Province (Argentina), and *G. jeffersonii* (Rees, 1993) collected in the Jurassic sediments of Botany Bay (Antarctic Peninsula; see also Rees and Cleal, 2004). However, the description of the last species is based on 15 highly fragmentary specimens and the presence of intercalary elements has not been so far reported (Cazaubon, 1947). *Goepertella jeffersonii* also has pinnules that match our specimens in overall shape, but differ in that they are slightly smaller, the rachises of primary segments and pinnae are thinner and the basalmost pinnule is borne on the acroscopic side of the pinna (versus the basiscopic in our studied specimens). Likewise, the rachial pinnules in *G. jeffersonii* are slightly narrower and overlap with the basal pinnules of the neighbouring pinnae, something that was not observed in our specimens (Rees 1993, pl. 1).

Several foliar morphological features such as the thickness of primary segment rachises, angle of insertion and pinnae spacing, are also shared among *G. unicyclica* and other Jurassic species: G. *herbstii* from Estancia La Juanita (Santa Cruz province, Argentina; Arrondo, 1972) and *G. diazii* from Alicurá (Nestares Formation. Neuquén province, Argentina) (Arrondo, 1972; Arrondo and Petriella, 1980, 1982). However, these two species are characterized by the presence of more than one rachial element being disposed between two successive pinnae and, therefore, are clearly distinguished from the specimens described here. The good preservation of the *G. unicyclica* fragments studied also made it possible to observe leaf microscopic characters in detail. In this regard, it was possible to corroborate the presence of ribs or grooves along the rachis of the primary segment and pinna rachis, a character that is widely found in the species of the genus and has also been described for *G. microloba*, *G. neuqueniana*, *G. herbstii*, *G. stipanicicii*, *G. varida* and *G. memoria-watanabei*. Likewise, the presence of trichomes on the laminar tissue, pinna rachis and primary segments is recorded here for the first time, on the basis of small rounded to slightly elongated depressions observed to be uniformly distributed on the surface being interpreted here as trichome bases (Figs. 6C, 8F). Interestingly, there is no other reference to the presence of indument in *Goeppertella*, except for the mention (without illustration) of scale bases on the rachises of primary segments of *G. jeffersonii* (Rees, 1993).

#### Reproductive morphology:

Previous records of fertile specimens of *Goeppertella* are scarce and extremely fragmentary (Herbst, 1966, 1992, 2000). Therefore, the fossils analyzed in this work constitute the largest collection of fertile leaf fragments known to date that also have good detail of micromorphological features such as the trichomes bases and the sporangia annuli preserved.

The sori of *G. unicyclica* resembles the ‘fructifications’ described for *G. stipanicicii* from the Late Triassic of Neuquén in relation to shape and dimensions (Herbst, 1992), though no further comparisons can be made as the distribution of sporangia within these structures of *G. stipanicicii* are not known. Additionally, some similarities between *G. unicyclica* with other Jurassic species were observed in terms of reproductive structures. The arrangement, shape and dimensions of the sori of our specimens resemble those described for fragments of *G. neuqueniana* (Early Jurassic, Neuquén Province), although *G. neuqueniana* differed in having between 8 and 14 sporangia per sorus (Herbst, 1966). The scheme published by Herbst (1992) shows that the sporangia of *G. neuqueniana* seemed to be organized similarly to *G. unicyclica* (i.e. sporangia in a single ring and less than 10 sporangia per sori), and the author described this arrangement as a rosette (radial arrangement).

The ovate sori of about 0.7-1 mm across described for *G. woodii* from Jurassic of Antarctic Peninsula (Rees, 1993) also have some similarities with our specimens. Although the article described *G. woodii* as having sori composed of 10 sporangia, these details cannot be made out based on the images provided of the fertile fronds (Rees op. cit., plate 2, fig. 1, 3-4).

The best-preserved record that exists of the reproductive structures of the genus, up to the present work, is that of the sporangia found in fragments of *G. taverai* from the Latter Triassic of Chile (Las Breas Formation) (Herbst, 2000). This species is described as having circular sporangia 2-1.6 mm in diameter located along the middle veins of pinnules. Likewise, it shows impressions of the cells of the annulus with an uncertain position but, apparently, forming a continuous structure (plate 4, fig. 23, op cit.). The diameter of the sporangia described for *G. taverai* is of a scale that is more similar to the sori observed in this work (about 0.9-1.1 mm) than for sporangia (about 0.2 mm). Likewise, the sporangia found in our specimens present interrupted annuli, with cells 0.03-0.05 mm wide. These striking differences may be due to a misinterpretation of the reproductive structures of *G. taverai* due to poor preservation, and it appears likely that what were described as sporangia for *G. taverai* actually represent sori. However, this is difficult to corroborate without accessing the original material because the photograph published in the aforementioned article is not to scale.

The arrangement of the sori on the lamina and the characters of sporangia within the sori could provide indirect evidence of their development (Schölch, 2000). The specimens in which a smaller number of sori was observed, the sori were found associated with the basal half of the pinnules, while in those fragments where a higher density of sori was observed, they covered more than half of the pinnula. A similar distribution was described for *G. woodii* (Rees, 1993) and would coincide with acroscropic maturation of these sets of sporangia. Likewise, the author observed that sori group together giving an appearance of elongated sori. Similarly, we observed specimens with two types of sori arrangement, isolated or superimposed, which could correspond to different stages of maturation of the fertile pinnula. It should be noted that in those cases where overlapping sori were observed, their counting and identification of the insertion areas of the sporangia was difficult.

Inside the sori, the sporangia of *G. unicyclica* have approximately the same size and shape, which could indicate their simultaneous maturation sequence. This kind of maturation, together with the arrangement of the sori in a single row, was identified as primitive for dissected species of other genera of the family such as *Dipteris* (Armour, 1907) and contrasts with the mixed maturation described for most of the Dipteridaceae (Schölch, 2003).

### Phylogenetic and affinity analysis

The incorporation of *Goeppertella* unicyclica to the taxon sampling of Choo and Escapa (2018) did not contribute to substantially resolve the relationships between the fossil taxa after the analysis of maximum parsimony. In the strict consensus tree, *G. unicyclica* forms a polytomous clade with the current species, sister to all extinct species **[see****Supporting Information 1****]**. However, the exquisite preservation of characters considered diagnostic for both Matoniaceae and Dipteridaceae made it possible to evaluate the affinity of *Goeppertella* with these families.

**Figure 9.**
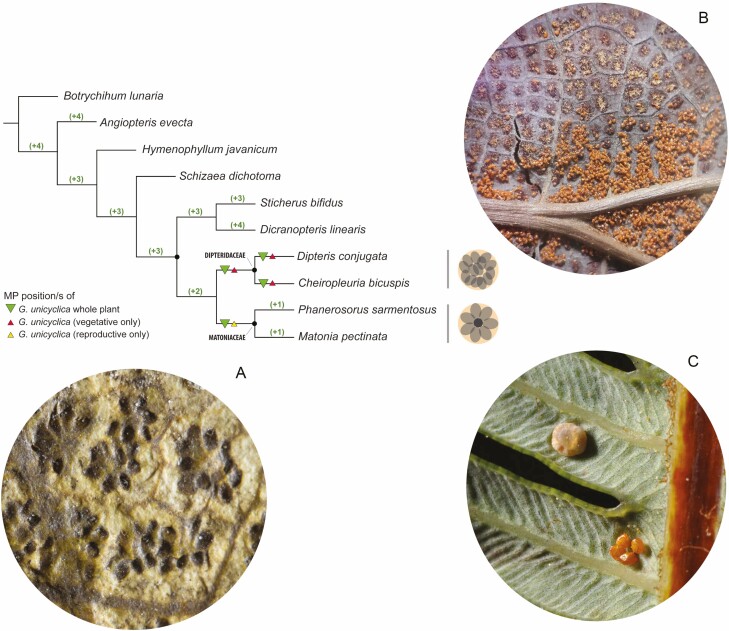
Results for *Goeppertella unicyclica* from combined backbone analyses using maximum parsimony. The position of the fossil species was estimated based on 52 morphological characters. Green triangles indicate the most parsimonious positions for this analysis. Green numbers indicate the extra steps required to place the fossils on these nodes. Red triangles indicate the optimal position of *G. unicyclica* if it is calculated exclusively on the vegetative characters and the yellow triangle indicates its position on the base of the reproductive characters. The drawings to the right of the tree and the images outline the most frequent arrangement of the sporangia within the sorus of Matoniaceae and Dipteridaceae in general and G. unicyclica in particular. (A) Goeppertella unicyclica, herein described. (B) Dipteris conjugata (Ph Yañez A.). (C) *Matonia foxworthyi* (Ph J.F. Barcelona & P.B. Pelser, from Nickrent *et al*., 2006 onwards).

In this sense, analysing the most parsimonious (MP) positions of *Goeppertella unicyclica* using the backbone of only extant Gleicheniales and the whole matrix (vegetative+reproductive characters), we found that the fossil species takes four equally parsimonious positions, with 76 steps. The positions of the genus vary among two positions as crown in Dipteridaceae, together with a position as stem of Dipteridaceae and one as stem of Matoniaceae (Fig. 9, green triangles). Analysing the affinity of the genus with other nodes in the tree, it is possible to see that the position of *Goeppertella* within the Matoniaceae crown group requires only one extra step, while its position as sister of Dipteridaceae+Matoniaceae, two extra steps. Finally, the position of Goeppertella with all the other nodes in the tree requires 3-4 extra steps.

When the position of *Goeppertella unicyclica* is analyzed exclusively with the vegetative characters, the species occupies the same positions within and sister to Dipteridaceae, but is not placed as sister to Matoniaceae. On the other hand, when the reproductive characters are analyzed alone, the fossil species takes a single MP position, as sister to Matoniaceae.

## Discussion

Taken together, the vegetative and reproductive morphology exhibited by *Goeppertella*, and represented here by the Jurassic species *G. unicyclica*, shows a combination of characters that is unique in the context of extant and fossil Dipteridaceae. Most representatives of the family have fronds that are characterized by a variable number of sori arranged within areoles, bearing numerous irregularly crowded sporangia (Oishi and Yamasita, 1936; Herbst, 1992; Gnaedinger and Zavattieri, 2021). Furthermore, some species of *Thaumatopteris* (Herbst, 1965; Lu *et al.*, 2021), *Dictyophyllum* (Pott *et al.* 2018) and *Hausmannia* (Stockey *et al*., 2006) show sporangia that seem to be distributed without forming well-defined sori, and following an acrosticoid morphology.

In addition to the mentioned similarities with other poorly preserved or fragmentary *Goeppertella* species **[see****Supporting Information 3—Table S2**], a scarce number of dipteridaceous species in other genera exhibit some morphological features that resemble those described for *G. unicyclica*. The uniseriate radial arrangement of sporangia was previously mentioned from the late Triassic *Dictyophyllum falcatum* (Kon’no, 1968), a species that was recently combined to the genus *Sewardalea* (Choo and Escapa, 2018). Although the arrangement of the sporangia seems to be similar to the Jurassic species described here, the sori were only illustrated through a diagram (Fig. 1G, H; Kon’no, 1968), and the author does not refer in his description to the presence of a receptacular area. Other *Sewardalea* species were also described from fertile impressions, but they were either described with the typical ‘crowded’ arrangement in the sori (e.g., *Sewardalea exile* and *S. spiralis* in Nathorst, 1906) or was not detailed (Zeiller, 1903; Sierotin, 1962; Potonié, 1967; Van Konijnenburg-van Cittert *et al*., 2020). It is interesting to note that *Sewardalea*, together with *Thaumatopteris*, have been recovered as a basal grade in the evolution of Dipteridaceae (Choo and Escapa, 2018), which can explain the presence of these plesiomorphic conditions. A detailed review of these forms, together with *Goeppertella*, will be crucial in order to understand the sequence of morphological transformation that occurred in the early evolution of this lineage.

*G. unicyclica* shows sori with a regular number of seven-eight sporangia forming a single ring disposed around a central insertion zone, further resembling the soral morphology of Matoniaceae. In Matoniaceae, the sporangia are closely arranged in one (occasionally two or four) concentric rows around a central area named receptacle or placenta (Skog and Litwin 1995; Klavins *et al*., 2004), which is vascularized by multiple veins and is often delineated by a well-defined outline (Ash, 1972; Kato & Iwatsuki, 1985; Passalia *et al*., 2018). Likewise, it has been suggested that the receptacle is probably homologous to the indusia, described for some extinct genera within Matoniaceae (Skog and Litwin, 1995; Klavins *et al*., 2004). The fossil record shows many examples of matoniaceous species that have similar reproductive morphology to *Goerppertella*, including *Phlebopteris* (Weber, 2008; Barbacka *et al*., 2018; Van Konijnenburg-van Cittert *et al*., 2020), *Konijnenburgia* (Kvaček & Dašková, 2010), *Matonia* (Barbacka *et al*., 2016)*, Matonidium* (Zeba-Bano & Bose, 1981) and *Aninopteris* (Givulescu and Popa, 1998). Despite the similarities in sori morphology between *G. unicyclica* and Matoniaceae, the density and distribution of sori in the Patagonian species show a typical dipteridaceuous arrangement. Sori in completely developed fronds in Dipteridaceae are usually distributed across the entire surface of the lamina, while in Matoniaceae the sori are usually organized in rows on both sides of the midveins. One of the few exceptions is the recent new genus of Dipteridaceae, *Patagoniapteris*, for which a single row of sori on each side of the primary veins was described (Gnaedinger and Zavattieri, 2021).

The mosaic of vegetative and reproductive characters, evidenced in the context of this manuscript, also has implications from the phylogenetic point of view. Choo and Escapa (2018) developed a phylogenetic study where *Goeppertella* was scored following two schemes: (i) based exclusively on the observed reproductive and vegetative characters and (ii) based on the frond architecture hypothesis originally proposed by Rees (1993). In the first case, the *Goeppertella* species were not recovered as monophyletic and form part of a large basal polytomy in the family, which includes the species belonging to several other fossil genera as terminals (e.g. *Thaumatopteris*, *Sewardalea* and *Dyctiophyllum*). Following the second scheme, *Goeppertella* is recovered as a monophyletic group, but it is also located in a similar basal polytomy. However, by removing *Goeppertella* from the analysis, a basal grade in the phylogeny of the family is recovered, which includes *Thaumatopteris* as the result of the first divergence, and *Sewardalea* as the second divergence (Fig. 2; Choo and Escapa, 2018). It is interesting to note that regardless of whether architectural characters are considered or not, *Goeppertella* seems to introduce conflict in the analysis, and following our interpretation this is due to a clear conflict of characters. This is supported by the backbone analysis carried out in this study, in which *Goeppertella* occupies alternative positions as stem and crown of Dipteridaceae, and as stem of Matoniaceae (Fig. 9). However, when analyzing the affinity based exclusively on reproductive characters, and exclusively on vegetative characters, the results show differences that evidenced the origin of the character conflict. While considering just vegetative features *Goeppertella* shows affinity with Dipteridaceae, mainly due to the characteristics of its reticulate venation; the reproductive characters analyzed in isolation show affinity with the base of Matoniaceae (Fig. 9). In this sense, the vegetative-reproductive mosaic shown by *Goeppertella*, and illustrated in this work, provides further understanding of the origin of the previously postulated phylogenetic uncertainty.

To summarize, *Goeppertella* has phylogenetic instability and takes different positions near to the base of the family Dipteridaceae (Choo and Escapa, 2018) due to the combination of derived vegetative and plesiomorphic reproductive characters. This position is also evidenced in the characteristics of its venation which, despite being anastomosed, shows a degree of anastomosis that is notably simpler than in other representatives of the family (Ôishi and Huzioka, 1941; Arrondo and Petriella, 1982; Herbst, 1993; Rees 1993). This allows us to hypothesize that, if the morphological organization shown by *Goeppertella* is a early diverging form in the evolution of the Dipteridaceae, there would have been two changes during the evolution of the family: one in the distribution of the sori, from an ordered distribution to a disordered one, and one in the anastomosed venation, from forms with lower to higher degrees of areolation. In her study about morphological traits of the family, Choo (2017) discussed the relationship between sporangia distribution, venation and lamina dissection, and concluded that the presence of such characters would be correlated. According to the author, dissected fronds typically have small pinnae, which do not require a complex irrigation system (they were found to be strongly correlated with free veins). Following this same line of thought, the increase in the complexity of its veining as a consequence of the fusion of the lamina would have allowed a change in the distribution of the sporangia towards more disordered and dispersed configurations.

Further progress in testing this hypothesis will require new studies expanding the character sampling, also including continuous characters as part of the analysis, since many relevant features (e.g. spore size) are varying in this scale. Also, since *Goeppertella* is introducing conflict in the basal nodes of Matoniaceae and Dipteridaceae, new analyses will also require an expanded taxon sampling in relation to that presented by Choo and Escapa (2018), since the inclusion of more fossils of the families Matoniaceae and Gleicheniaceae will be required.

## Remarks

As we discussed, *Goeppertella unicyclica* shows a combination of features, with vegetative morphology showing affinities with fossil and extant Dipteriaceae, contrasting with the reproductive morphology being more comparable with the sister family, Matoniaceae. In this context, we consider *Goeppertella* as a member of the family Dipteridaceae since we interpret most shared features with Matoniaceae (e.g. sporangia arrangement) as plesiomorphic conditions for the family, as is supported by the multiple fossil occurrences of this morphology. In contrast, most shared features with Dipteridaceae (e.g. venation) represent apomorphies for the group. Thus, *Goeppertella* would represent a basal genus in Dipteridaceae, considering the venation characters as the most important in order to define the family. In order further explore this hypothesis, it is crucial to develop a total evidence phylogenetic analysis, including fossil and extant representatives of Matoniceae, Dipteridaceae and Gleicheniaceae.

## Supporting Information

The following additional information is available in the on- line version of this article—

**SI1**. Strict consensus tree obtained from the modified data set of Choo and Escapa (2018). Taxa are colored to indicate outgroups (black), Matoniaceae (red), Dipteridaceae (green) and new species here described (pink). Fossil specimens are indicated by italics.

**SI2**. **Table 1.** Localization and age of *Goeppertella* species for comparisons with *G. unicyclica*. Original description written in bold.

**SI3**. **Table 2.** Morphological comparisons of *Goeppertella* species with *G. unicyclica*. Original description written in bold. Abbreviations for character 11: bp, Between pinnae; bpp, between pairs of pinnae. Match with *G. unicyclica* in relation to discrete characters: light gray = partial match; dark gray = exact match. Match with *G. unicyclica* in relation to continuous characters: light gray = the character range of *G. unicyclica* overlap between 50% and 75%; dark gray = the character range of *G. unicyclica* overlap more than 75%. Characters: Fragments of PS 1, wide of primary segments (cm); 2, length of primary segments (cm); 3, ribs in rachis of primary segments; 4, sulcus in rachis of primary segments; 5, wide of rachis of primary segments; 6, length of rachis of primary segments; 7, trichome bases on rachis of primary segments; 8, pinnae arrangement; 9, angle between PS and pinnae; 10, pinnae insertion; 11, pinnae insertion intervals (cm); 12, wide of pinnae (cm); 13, length of pinnae (cm); 14, degree of pinnae dissection; 15, ribs in rachis of pinnae; 16, wide of pinnae rachis; 17, length of pinnae rachis; 18, trichome bases on rachis of pinnae; 19, diameter of trichome bases (mm); 20, pinnules arrangement; 21, angle between pinna-rachis and pinnules (midvein); 22, shape of pinnules; 23, pinnules insertion; 24, position of basalmost pinnule; 25, approximate pinnules proportions; 26, length of pinnules (cm); 27, wide of pinnules (cm); 28, margin of pinnules; 29, apex of pinnules; 30, trichoma bases on pinnules; 31, type of subsidiary elements sensu Arrondo and Petriella 1982; 32, foliar wing (or rachial lamina s. Rees 1993); 33, number of subsidiary elements between pinnae; 34, shape of subsidiary elements between pinnae; 35, arrangement of subsidiary elements with respect to pinnae; 36, cusp (Arrondo & Petriella 1982); 37, insertion of subsidiary elements; 38, overlapping of subsidiary elements with pinna; 39, subsidiary elements insertion intervals (cm); 40, wide of subsidiary elements (mm); 41, Length of subsidiary elements (mm); 42, axilar element or pinnule; 43, wide of midvein; 42, angle between midvein and lateral vein; 45, midvein reach; 46, marginal vein; 47, secondary veins; 48, meshes; 49, amount of meshes order; 50, diameter of major areola (mm); 51, free-included veinlets; 52, diameter of sori (mm); 53, shape of sori; 54, confluence of sori; 55, position of sori; 56, ordering of sori; 57, N° sporangia per sorus; 58, diameter of sporangium (mm); 59, position of ring sporangium; 60, N° cell of ring sporangium annulus; 61, apparent maturation of sporangia.

plad007_suppl_Supplementary_Information

## Data Availability

The data set generated during the current study are available from http://morphobank.org/permalink/?P4530.

## Notes

**Evolution & Diversity. Chief Editor:** Jeremy Beaulieu

## Appendix

**Table:** **Appendix 1.** List of morphological characters used in the phylogenetic analysis

No.	Character description
	Vegetative
1	Rhizome: Upright (0), Creeping (1)
2	Rhizome indument: Absent (0), Present (1)
3	Rhizome indument type: Uniseriate hairs (0), Bristles [multiseriate but not flattened] (1), Scales (2)
4	Rhizome vasculature: Protostele (0), Solenostele (1), Dictyostele (2)
5	Solenostele: Single (0), Polycyclic (1)
6	Stipe vasculature: Single bundle (0), Multiple bundles (1)
7	Stipe vasculature shape: C-shaped (0), Omega (1), Ring (2), Solid central bundle (3)
8	Rachial architecture: Branching rachis resulting in two or more axes of growth (0), unbranched rachis, i.e., one axis of growth (1)
9	Initial rachial dichotomy: Isotomous (0), Anisotomous (1)
10	Subsequent rachial dichotomies: Isotomous (0), Anisotomous (1)
11	Directionality of anisotomous dichotomies: Catadromous (0), Alternate (1)
12	Dichotomizing axis reduced to rachial bud: Never (0), Yes (1)
13	For pedate fronds, number of primary segments on each rachial arm: 1–2 (0), 3–6 (1), 7–12 (2), 13–20 (3), 20–50 (4), >50 (5)
14	For pedate fronds, degree of laminar fusion between primary segments: None (0), Up to 1/3 fused (1), 1/3–2/3 fused (2), 2/3 to almost fully fused (3), Fully fused (4)
15	For pedate fronds with simple veins, degree of laminar fusion between secondary veins: None (0), Up to 1/3 fused (1), 1/3–2/3 fused (2), 2/3 to almost fully fused (3), Fully fused (4)
16	For pedate fronds, degree of fusion between tertiary veins: None (0), Up to 1/3 fused (1), 1/3–2/3 fused (2), 2/3 to fully fused (3)
17	For pedate fronds with simple primary veins, primary segment shape: Linear (0), Lanceolate–oblanceolate (1)
18	For pedate fronds, maximum width of frond: <50 cm (0), 50–100 cm (1), >100 cm (2)
19	For pedate fronds without free primary segments, overall leaf lamina bipartite: No (0), Yes (1)
20	For pedate fronds, lamina dissecting within primary segment between branched primary veins: No (0), Yes (1)
21	Venation type: Free (0), Anastomosing without free-ending veinlets (1), Anastomosing with free-ending veinlets (2)
22	For free-veined taxa, ultimate veins showing multiple sympodial dichotomizing venation pattern: No (0), Yes (1)
23	Degree of anastomosis: Free/Furcate (0), areoles 1st order (1), areoles 2nd order (2), areoles order >2 (3).
24	Secondary and tertiary veins forming regular orthogonal areoles: Yes (0), No (1)
25	Arrangement of ultimate areoles: Areoles only found locally on frond (0), Meshwork of areoles throughout leaf lamina (1)
26	Size of ultimate areoles: <1 mm^2^ (0), 1–3 mm^2^ (1), >3 mm^2^ (2)
27	Proportion of ultimate areoles with free-ending veinlets: <50% (0), >50% (1)
28	Areolar free-ending veinlets branched: Mostly not branched (0), Mostly branched (1)
29	For pedate fronds, primary veins: Simple (0), Dichotomizing (1)
30	For pedate fronds with dichotomizing primary veins, primary veins anastomosing: Never (0), Yes (1)
31	For pedate fronds, secondary veins: Simple (0), Dichotomizing (1)
32	Stomata type: Anomocytic (0), Paracytic (1), Cyclocytic (2), Other (3)
	REPRODUCTIVE
33	Sterile/fertile dimorphy: Fronds monomorphic (0), Fronds dimorphic (1)
34	Degree of dimorphism: Holodimorphic (0), Hemidimorphic (1)
35	Position of sporangia: On leaf lamina (0), Along leaf margin (1)
36	Percentage of frond lamina covered by sporangia: <50% (0), 50–90% (1), >90% (2)
37	Shape of sori: Circular to oval (0), Oblong to linear (1)
38	Arrangement of sporangia within sorus: In a single row (0), In multiple rows (1)
39	Presence of prominent central receptacle in sorus: No (0), Yes (1)
40	Number of sporangia per sorus: Very few 3–8 (0), Few 9–15 (1), Many >15 (2)
41	Sporangial maturation: Simultaneous (0), Mixed (1)
42	Diameter of sporangial capsule: <200 μm (0), 200–400 μm (1), 400–600 μm (2), >600 μm
43	Number of cell files in sporangial stalk: 0 (0), 1 (1), 2 (2), 3 (3), 4 (4), >4 (5)
44	Ratio of length of sporangial stalk to diameter of sporangial capsule: ≤1 (0), >1 (1)
45	Presence of annulus: Absent (0), Present (1)
46	Annulus type: Patch (0), Ring (1)
47	Annulus orientation: Lateral (0), Oblique to vertical (1)
48	Ring-type annulus interrupted at stalk: No (0), Yes (1)
49	(for Gleicheniales) Spore size: <30 μm (0), 30–50 μm (1), >50 μm (2)
50	Spore shape: Trilete (0), Monolete (1)
51	Indusium: Absent (0), Present (1)
52	Indusium peltate: No (0), Yes (1)
