# Supplementary material for: Fertile Goeppertella from the Jurassic of Patagonia: mosaic evolution in the Dipteridaceae-Matoniaceae lineage
Source: AoB Plants. 2023 Jun 7;15(4):plad007. doi: 10.1093/aobpla/plad007 (PMC10324646; doi:10.1093/aobpla/plad007)
Supplement: plad007_suppl_Supplementary_Information [file plad007_suppl_supplementary_information.pdf]

SI1. Strict consensus tree obtained from the modified data set of Choo and Escapa (2018). Taxa are colored to indicate outgroups (black), Matoniaceae (red), Dipteridaceae (green) and new species here described (pink). Fossil specimens are indicated by italics.

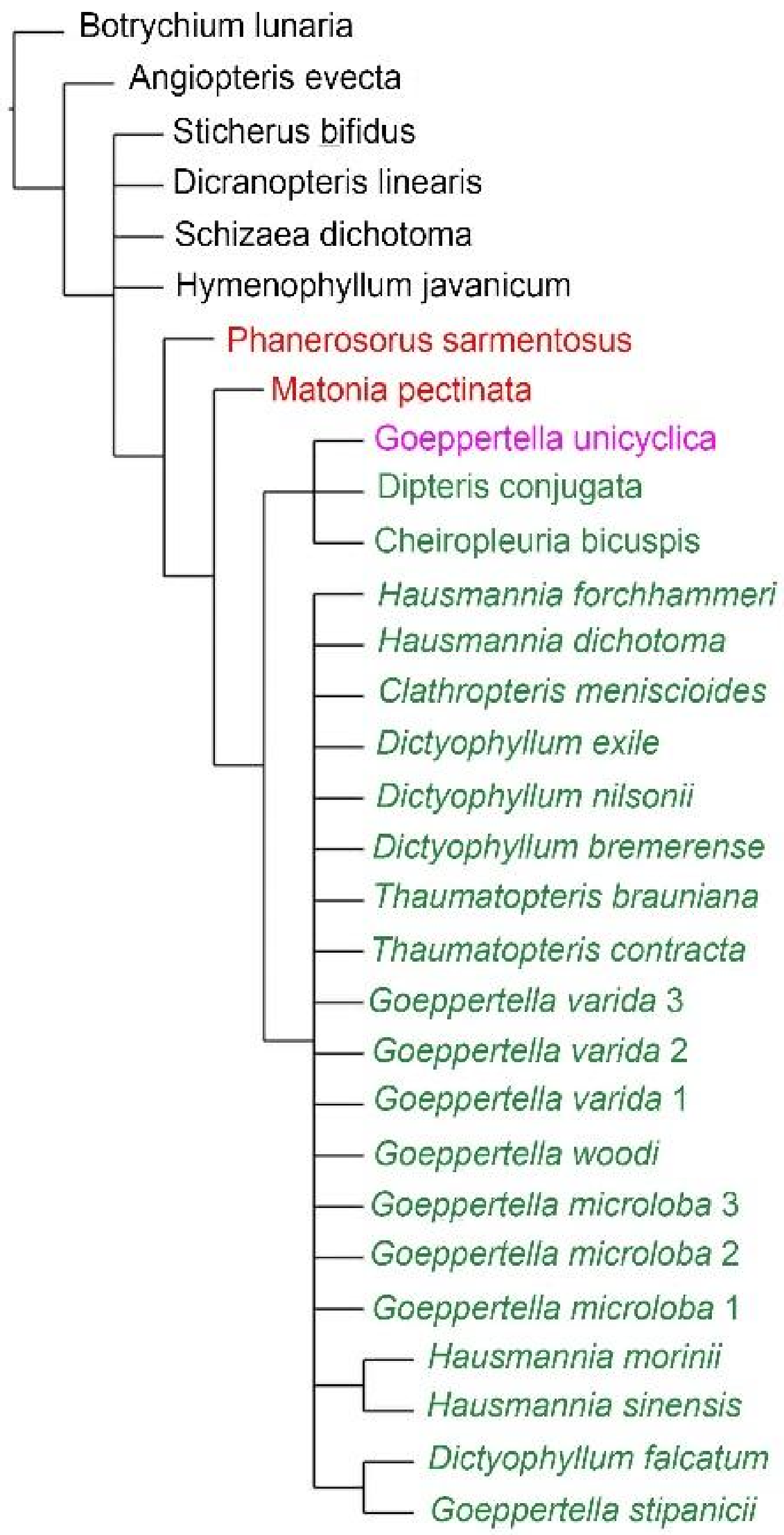

SI1. Table 1. Localization and age of *Goeppertella* species for comparisons with *G. unicyclica*. Original description written in bold.

	**Locality and area**	**Formation and age**	**RESOURCE**
G. unicyclica	Cerro Bayo, Chubut	Unnamed stratigraphic unit, lateral equivalent of Las Leoneras Formation (Early Jurassic)	This article
G. frengelliana	Cordón de Esquel, Arroyo Martinez, Cerro Groeber, Chubut.	Middle-upper Jurassic	**Cazaubón (1947)**.
G. macroloba	Puesto de C. Meschio, Sierra<br>Pampa de Agnia, Chubut.	Cerro Puntudo Alto Formation (Middle Jurassic).	**Herbst (1964,** 1968**)**
	Botany Bay, Antartica.	Monte Flora Formation (Middle Jurassic).	Morel (1994).
	Alicurá region, Río Negro.	Nestares Formation (Lower Jurassic).	Sagasti et al. (2019).
G. neuqueniana	Cerro Mesa, Cañadón de Piedra Pintada, Neuquén.	Piedra Pintada Formation (Lower Jurassic).	**Herbst (1966,** 1968, 1992**)**, Stipanicic y Bonetti (1970), Morel (1994).
	Alicurá region, Río Negro.	Nestares Formation (Lower Jurassic).	Sagasti et al. (2019).
G. herbstii	Estancia La Juanita, Santa Cruz.	Grupo Bahía Laura, Chon-Aike? Formation (Jurassic).	Arrondo (1972), **Arrondo & Petriella (1982).**
G. diazzi	Alicurá, Neuquén.	Nestares Formation (Lower Jurassic-Hettangian).	**Arrondo & Petriella** (1980, **1982**), Morel et al. (2013).
G. stipanicicii	Paso Flores, Neuquén.	Paso Flores Formation, (Upper Triassic).	**Herbst (1993),** Morel et al. (1999).
	Alicurá, Neuquén.	Nestares Formation (Lower Jurassic-Hettangian).	Sagasti et al. (2019).
G. jeffersonii	Botany Bay and Botany Bay, Antarctic Peninsula.	Champ Hill Formation (Early-upper Jurassic).	**Rees (1993)**, Rees & Cleal (2004).
G. woodii	Botany Bay and Botany Bay, Antarctic Peninsula.	Champ Hill Formation (Early-upper Jurassic).	**Rees (1993).**

G. taverai	Punta del Viento, Chile.	Las Breas (Upper Jurassic)	**Herbst (2000).**
***Goeppertella* sp.**	Valle de Las Leñas, Neuquén.	El Freno (Lower Jurassic)	Lanés et al. (2013)
G. varida	Nariwa, Japan.	Triassic	**Oishi (1940).**
G. memoria-watabei	Hongäy, Vietnam.	Estratos de Tonkin (Upper Triassic)	**Oishi y Huzioka (1941)**, Arrondo & Petriella (1982).
G. microloba	Germany, Sweden, Vietnam.	Triassic-Jurassic	Schenk (1867); **Oishi & Shamasita (1936)**; Arrondo & Petriella (1982).
	French Indochina	Late Triassic	Zeiller (1903)
	Odrowąż, Poland.	Zagaje Formation (Early Jurassic).	Barbacka et al. (2010).
	Guangdong, China.	Xiaoping Formation (Upper Triassic)	Zhou et al. (2016).

SI3. Table 2a. Morphological comparisons of *Goeppertella species* with *G. unicyclica*. Original description written in bold. Abbreviations for character 11: bp, Between pinnae; bpp, between pairs of pinnae. Match with G. unicyclica in relation to discrete characters: light gray = partial match; dark gray = exact match. Match with *G. unicyclica* in relation to continuous characters: light gray = the character range of *G. unicyclica* overlap between 50% and 75%; dark gray = the character range of *G. unicyclica* overlap more than 75%. Characters: Fragments of PS 1, wide of primary segments (cm); 2, length of primary segments (cm); 3, ribs in rachis of primary segments; 4, sulcus in rachis of primary segments; 5, wide of rachis of primary segments; 6, length of rachis of primary segments; 7, trichome bases on rachis of primary segments; 8, pinnae arrangement; 9, angle between PS and pinnae; 10, pinnae insertion; 11, pinnae insertion intervals (cm); 12, wide of pinnae (cm); 13, length of pinnae (cm); 14, degree of pinnae dissection; 15, ribs in rachis of pinnae; 16, wide of pinnae rachis; 17, length of pinnae rachis; 18, trichome bases on rachis of pinnae; 19, diameter of trichome bases (mm); 20, pinnules arrangement; 21, angle between pinna-rachis and pinnules (midvein); 22, shape of pinnules; 23, pinnules insertion; 24, position of basalmost pinnule; 25, approximate pinnules proportions; 26, length of pinnules (cm); 27, wide of pinnules (cm); 28, margin of pinnules; 29, apex of pinnules; 30, trichoma bases on pinnules; 31, type of subsidiary elements sensu Arrondo & Petriella 1982; 32, foliar wing (or rachial lamina s. Rees 1993); 33, number of subsidiary elements between pinnae; 34, shape of subsidiary elements between pinnae; 35, arrangement of subsidiary elements with respect to pinnae; 36, cusp (Arrondo & Petriella 1982); 37, insertion of subsidiary elements; 38, overlapping of subsidiary elements with pinna; 39, subsidiary elements insertion intervals (cm); 40, wide of subsidiary elements (mm); 41, Length of subsidiary elements (mm); 42, axilar element or pinnule; 43, wide of midvein; 42, angle between midvein and lateral vein; 45, midvein reach; 46, marginal vein; 47, secondary veins; 48, meshes; 49, amount of meshes order; 50, diameter of major areola (mm); 51, free-included veinlets; 52, diameter of sori (mm); 53, shape of sori; 54, confluence of sori; 55, position of sori; 56, ordering of sori; 57, N° sporangia per sorus; 58, diameter of sporangium (mm); 59, position of ring sporangium; 60, N° cell of ring sporangium annulus; 61, apparent maturation of sporangia.

	G. unicyclica	***G. frengelliana***	***G. macroloba***	***G. neuqueniana***	***G. herbstii***	***G. diazzi***
**Ch1**	>8.66	md	18	12-**25**	**>20**	**25**
**Ch2**	>16.3	md	12	6**-16**	**>23**	**30**
**Ch3**	Present (3)	md	Present	Present **(1)**	Present	md
**Ch4**	Absent	**Present**	md	md	Present	md

**Ch5**	2.26-4.75	**2.5-4.5**	**0.8-2**	0.02-**3.5-4**	**2-3**	**5**
**Ch6**	>16.3	**2.1-16**	md	md	md	md
**Ch7**	Present	md	md	md	md	md
**Ch8**	Subopposite/Alternate	**Subopposite**	Subopposite/**Alternate**	**Opposite**/Alternate	**Opposite**/Subopposite	**Subopposite**
**Ch9**	52°-79°	md	**45°-50°**	40°-45°-60°-**65°**-70°-**85°**	50°-80°	**90°**
**Ch10**	Sessile	**Slightly petioled**	md	Sessile	md	md
**Ch11**	2-3.7 (bpp), 0.8-1.9 (bp)	md	2.4-2.8 (bpp), **0.4 (bp)**	0.6-**1.5-2.8** (bpp), 0.2 (bp)	2.7-**3-4** (bpp)	**3** (bpp)
**Ch12**	1.44-5.01	6	0.6-**3.5**	**0.5-2**	4-**10**	**4.5**
**Ch13**	14.8	1.6-**2.25-4.7**	5-**9.5-**10	**>8-9**	**<15**	**12.5**
**Ch14**	>3/4	3/4	3/4	>3/4	md	md
**Ch15**	Absent	**Thin stries**	**Smooth striation**	md	md	md
**Ch16**	0.29-2.4	**1**	**0.6-**3	0.5-**1**	1-1.5	md
**Ch17**	14.8	**>22.5-24-34**	md	md	md	md
**Ch18**	Oval pits	Present	md	md	md	md
**Ch19**	0.06-0.1	md	md	md	md	md
**Ch20**	Opposite to alternate	md	**Alternate**	**Opposite to alternate**	**Opposite (basal) / Alternate (distal)**	**Suboposite/Alternate**
**Ch21**	**-**	**40°-45°-80°-85°**	**45°-50°**-70°-80°-85°-90°	50°-**60°-80°**	**50°-80°**	70°-**90°**
**Ch22**	Lanceate/Falcate	Falcate	**Triangular/Falcate**	**Falcate**/Subfalcate	**Slightly falcate**/ Triangular	Straight to Falcate
**Ch23**	Sessile	md	Sessile	md	md	md

**Ch24**	Basiscopic	md	md	md	md	md
**Ch25**	md	md	**Basalmost smaller**	md	md	md
**Ch26**	0.67-3.57	>**1.5-**2.5	**1.5-2.5-2.7-**7.3	0.25-**0.8-1**	2-**3-5**	**1.5-2**
**Ch27**	0.62-2.08	**1.9**	**1.2-1.7-**2	0.15-**0.5**	**1.5-2**	**0.7**
**Ch28**	Entire	**Entire**	**Entire**/Crenate/Lobulated	**Entire**	**Entire**	**Irregularly undulated**
**Ch29**	Acute	**Acute**	Rounded/**Acute**	**Obtuse to sligtlhy acute**	**Obtuse**	**Obtuse/Acute**
**Ch30**	Trichome bases	md	md	md	md	md
**Ch31**	Intercalar	Absent/Intercalar	Intercalar	Intercalar	**Intercalar**	Intercalar
**Ch32**	Absent	Absent	**Present**	Present	md	**Present**
**Ch33**	1	md	md	**1(occassionaly 3)**	>1	md
**Ch34**	Deltate/auriculate	md	md	**Pecopterid**	**Triangular/Falcate**	md
**Ch35**	Subopposite/Alternate	md	md	md	Opposite	md
**Ch36**	1	md	1	1	**2**	**2-3**
**Ch37**	Sessile	md	md	md	md	md
**Ch38**	No	md	md	md	md	md
**Ch39**	9.97-20.73	md	md	md	md	md
**Ch40**	14.17	md	md	md	md	md
**Ch41**	8.35-13.01	md	10	6	15	**-**
**Ch42**	Absent	Absent	Absent	Absent	Absent	Absent

**Ch43**	0.2-0.7	md	1	md	md	md
**Ch44**	50°-78°	md	md	**75°-90°**	md	md
**Ch45**	Subapical/Apical	**Apical**	**Apical**	Subapical-**Apical**	md	md
**Ch46**	Absent	md	md	md	md	md
**Ch47**	Less obvious	md	md	md	md	md
**Ch48**	Polygonal/Rectangular	**Polygonal**	Polygonal/Rectangular	**Polygonal**	md	md
**Ch49**	3	md	**3**	**3**	**3**	**2-3**
**Ch50**	1.6-2.8	md	**4.5-5x3.5**	1.5-2	**8x6**	md
**Ch51**	Apparently present	**Absent**	Present	Present	md	md
**Ch52**	0.9-1.1	md	md	**1-1.2**	md	md
**Ch53**	Rounded	md	md	**Circular**	md	md
**Ch54**	Yes	**-**	**-**	**-**	**-**	**-**
**Ch55**	Both sides of the middle vein	md	md	**Both sides of the middle vein**	md	md
**Ch56**	Rosette	md	md	**Rosette**	md	md
**Ch57**	7	md	md	**8-14**	md	md
**Ch58**	0.2	md	md	md	md	md
**Ch59**	**-**	md	md	md	md	md
**Ch60**	11	md	md	**8**	md	md
**Ch61**	Acroscopic	md	md	md	md	md

SI3. Table 2b. Morphological comparisons of *Goeppertella* species with *G. unicyclica* (continuation).

	G. stipanicicii	***G. jeffersonii***	***G. woodii***	***G. taverai***	**Goeppertella sp.**
**Ch1**	8.25	md	>3	17	md
**Ch2**	>8.7	md	>120	18	md
**Ch3**	**Smooth or softly fluted**	md	md	md	md
**Ch4**	md	md	md	md	md
**Ch5**	0.15-**3**	**1.2**	md	**2**	1.5-2
**Ch6**	md	md	md	md	md
**Ch7**	md	**Present (scales)**	md	md	md
**Ch8**	**Subopposite/Alternate**	**Subopposite/Alternate**	**Subopposite/Alternate/Opposite**	md	md
**Ch9**	**40°-45°**-50°-70°-85°	**40°-80°**	**30°-75° (40°-60°)**	md	md
**Ch10**	md	md	md	md	md
**Ch11**	**0.1-2.5 (bpp),** 0.57 (bp)	**1.2-1.5 (bp)**	**1.1-4**	md	md
**Ch12**	**12-14-**24	md	md	**2.2**	md
**Ch13**	5-**11**	**5-10.5**	**8-10**	**8.5**	md
**Ch14**	md	**3/4**	**3/4**	**1/2 (pinnatifid)**	md
**Ch15**	md	md	md	md	md
**Ch16**	**1.5**	**0.1-0.5-1**	**0.2-1.3**	md	0.5-0.9
**Ch17**	md	md	md	md	md

**Ch18**	md	md	Present (oval pits)	md	md
**Ch19**	md	md	0.05-0.2	md	md
**Ch20**	Subopposite	**Subopposite / Opposite / Alternate**	**Subopposite / Opposite / Alternate**	md	md
**Ch21**	**45°**-70°-90°	**40°-50°-60°-70°-90°**	**40°-90°**	md	35 º -40º
**Ch22**	Triangular/Elongate/Falcate	**Wedge form / Falcate**	**Wedge / Squat proximally, narrow-falcate distally**	**Triangular**	md
**Ch23**	md	md	md	md	md
**Ch24**	md	**Acroscopic side**	md	md	md
**Ch25**	md	Smaller towards pinna apex	md	md	md
**Ch26**	**0.1-1.1**	**0.4-1.3**	**0.6-2.5**	md	1.5
**Ch27**	**0.5-0.6**	**0.2-0.8**	**0.5-1.3**	md	0.05
**Ch28**	Lobate	**Entire-occasionally lobed/denticulate**	**Entire**	md	md
**Ch29**	Terminal lobe	**Acute to subacute**	**Acute to subacute**	md	Subacuminate to rounded
**Ch30**	md	md	md	md	md
**Ch31**	**Intercalar**	**Intercalar**	**Intercalar**	**Intercalar**	md
**Ch32**	md	**Absent**	**Present**	md	md
**Ch33**	>1	md	Sometimes 1	md	1 (2?)
**Ch34**	=Pinnules	**=Pinnules**	md	**Triangular**	md

**Ch35**	md	**Subopposite**	md	md	md
**Ch36**	**1**	**1**	md	md	md
**Ch37**	md	**Sessile**	md	md	md
**Ch38**	md	**Yes**	md	md	md
**Ch39**	md	md	md	md	md
**Ch40**	md	**3-6**	**6-13**	**1.5**	1.2
**Ch41**	**10**	**5-7**	md	**2.5**	md
**Ch42**	md	md	md	md	md
**Ch43**	md	md	**0.1**	md	md
**Ch44**	md	**60°**	md	md	md
**Ch45**	**Subapical**	md	md	md	md
**Ch46**	md	md	**Present**	md	md
**Ch47**	Less obvious	md	md	md	md
**Ch48**	Polygonal	**Polygonal**	**Polygonal**	**Isodiametric(1° order)/Polygonal**	Polygonal
**Ch49**	md	md	md	**2**	3
**Ch50**	**0.8-1.2**	**0.5-1-3**	**0.25-0.5**	**3-4**	3.5-4.2
**Ch51**	md	**Sometimes**	md	**Present**	md
**Ch52**	1.2 x 0.9	md	**0.7-1**	md	md
**Ch53**	Slighlty oval	md	md	md	md

**Ch54**	md	md	**Yes**	md	md
**Ch55**	md	md	md	**Along the median veins**	md
**Ch56**	md	md	md	md	md
**Ch57**	md	md	>10	md	md
**Ch58**	md	md	**0.1**	**1.2-1.6**	md
**Ch59**	md	md	md	md	md
**Ch60**	md	md	md	md	md
**Ch61**	md	md	Acroscopic	md	md

SI3. Table 2c. Morphological comparisons of *Goeppertella* species with *G. unicyclica* (continuation).

	G. varida	***G. memoria-watabei***	***G. microloba***
**Ch1**	**18**	md	md
**Ch2**	md	**<30**	md
**Ch3**	**Present**	md	Present
**Ch4**	md	md	1.5-2
**Ch5**	**2-3**	**2**	md
**Ch6**	md	md	md
**Ch7**	md	md	md
**Ch8**	**Subopposite/ Alternate**	**Subopposite**	**Alternate**
**Ch9**	**90°**	**90°**	md
**Ch10**	md	md	md
**Ch11**	md	**4.5**	0.5-2
**Ch12**	md	**3**	0.3-0.6-2.5
**Ch13**	md	**<6**	5-10
**Ch14**	md	md	Pinnate
**Ch15**	md	md	md
**Ch16**	md	**1**	md
**Ch17**	md	md	md

**Ch18**	md	md	md
**Ch19**	md	md	md
**Ch20**	md	**Subopposite**	Alternate
**Ch21**	md	md	80°–**90°**
**Ch22**	**Deltoid**	Subrectangular to subtriangular	Ovate-**Lanceolate**
**Ch23**	md	md	Fused at the base
**Ch24**	md	md	md
**Ch25**	md	md	md
**Ch26**	<0.5	**1.5-2**	0.1-0.5-0.6-1.3
**Ch27**	md	**0.5-0.8**	0.1-0.3-0.5
**Ch28**	**Undulating**	**Undulating -** Wavy to slightly lobulated	Entire, sometimes slightly crenate
**Ch29**	**Round-**Obtuse to subacute	**Obtuse -** occasionally subacute	**Rounded** to acute
**Ch30**	md	md	md
**Ch31**	Intercalar and axilar	Present	Intercalar
**Ch32**	**Present?**	md	Present
**Ch33**	md	md	md
**Ch34**	md	md	Tongue-form/Subtriangular
**Ch35**	md	md	md
**Ch36**	md	>4	1
**Ch37**	md	md	md

**Ch38**	md	md	md
**Ch39**	md	md	md
**Ch40**	md	md	md
**Ch41**	md	md	md
**Ch42**	Present	md	md
**Ch43**	md	md	md
**Ch44**	md	**90°**	**90°**
**Ch45**	md	**Apical**	**Apical**
**Ch46**	md	md	md
**Ch47**	md	md	Obvious
**Ch48**	**Polygonal**	md	Polygonal
**Ch49**	md	md	2
**Ch50**	md	md	md
**Ch51**	md	md	Present
**Ch52**	**0.5**	md	md
**Ch53**	**Round**	md	Oblong
**Ch54**	md	md	Yes
**Ch55**	**Both sides of the midnerve and of the pinna-axis**	md	md
**Ch56**	md	md	md
**Ch57**	**5-9**	md	5-8

**Ch58**	md	md	md
**Ch59**	md	md	md
**Ch60**	md	md	md
**Ch61**	md	md	md
